# Absence of Metformin in Fetal Circulation Following Maternal Administration in Late Gestation Pregnant Sheep

**DOI:** 10.1007/s43032-024-01547-2

**Published:** 2024-04-23

**Authors:** Paul J. Rozance, Laura D. Brown, Stephanie R. Wesolowski

**Affiliations:** Department of Pediatrics, Perinatal Research Center, University of Colorado School of Medicine, Mail Stop F441, Aurora, CO 80045 USA

**Keywords:** Metformin, Pregnancy, Fetal exposure

## Abstract

In human pregnancy, metformin administered to the mother crosses the placenta resulting in metformin exposure to the fetus. However, the effects of metformin exposure on the fetus are poorly understood and difficult to study in humans. Pregnant sheep are a powerful large animal model for studying fetal physiology. The objective of this study was to determine if maternally administered metformin at human dose-equivalent concentrations crosses the ovine placenta and equilibrates in the fetal circulation. To test this, metformin was administered to the pregnant ewe via continuous intravenous infusion or supplementation in the drinking water. Both administration routes increased maternal metformin concentrations to human dose-equivalent concentrations of ~ 10 µM, yet metformin was negligible in the fetus even after 3–4 days of maternal administration. In cotyledon and caruncle tissue, expression levels of the major metformin uptake transporter organic cation transporter 1 (*OCT1*) were < 1% of expression levels in the fetal liver, a tissue with abundant expression. Expression of other putative uptake transporters *OCT2* and *OCT3*, and efflux transporters multidrug and toxin extrusion (*MATE)1* and *MATE2*were more abundant. These results demonstrate that the ovine placenta is impermeable to maternal metformin administration. This is likely due to anatomical differences and increased interhaemal distance between the maternal and umbilical circulations in the ovine versus human placenta limiting placental metformin transport.

## Introduction

Metformin is a leading medication for the treatment of Type-2 diabetes (T2D) in adults and is recommended as an alternative to insulin in women with T2D or gestational diabetes [1]. Metformin crosses the human placenta exposing fetal tissues to adult concentrations of the drug [2]. Importantly, fetal macaque, sheep, and rodent tissues express metformin transporters [3, 4] with abundant expression and metformin uptake in the fetal liver following direct fetal metformin exposure in sheep [3]. Concerningly, emerging studies in human cohorts report adverse effects in offspring exposed to intrauterine metformin including decreased birth weight and increased risk for childhood adiposity [1, 5, 6].

The mechanism by which metformin influences fetal growth is unknown and difficult to study in humans. The pregnant sheep is an attractive model because in utero measurements of fetal nutrient utilization can be performed. The objective of this study is to evaluate the utility of pregnant sheep as a model system to understand the effects of intrauterine metformin exposure.

## Methods

### Maternal Metformin Administration

Columbia-Rambouillet sheep were studied following Institutional Animal Care and Use Committee guidelines. Surgery occurred at ~ 125 gestational days (~ 147 days gestation length) using established methods [7, 8]. Metformin was administered > 3 days after surgery. For bolus dosing, 500 mg metformin (Sigma, #D1590959) was prepared in 5 ml saline and infused over 5 min into maternal femoral vein catheter. Maternal and fetal arterial blood samples were collected simultaneously at 0, 30, 60, 120, 240, 360, and 480-min. For continuous infusion, metformin was prepared in saline (83 mg/mL) and filtered continuously for 24- or 72-h into maternal femoral vein catheter at 1 mL/h to deliver 2000 mg over 24 h to match human-dosing. Maternal and fetal blood samples were collected before and 4 h after infusion and every 24 h. For oral administration, 2000 mg metformin was dissolved in 3L of drinking water and provided in the morning after the ewe was thirsted for 3 h. After water was consumed, typically ≤ 60 min, fresh water was provided ad libitum. Daily dosing occurred for 4 consecutive days. Maternal and fetal blood samples were collected before and 4 h after dosing. Plasma metformin concentrations were measured using HPLC [3].

### RNA Isolation and Real-time PCR

Caruncle (maternal facing) and cotyledon (fetal facing) tissues were obtained from 8 normal late gestation pregnancies [8] and 2 late gestation fetal liver samples [3]. RNA was isolated and used for qRT-PCR using previously described methods and primers following MIQE guidelines [3, 7]. Results were normalized to S15 (*RPS15*), which demonstrates consistent expression between these tissues, and expressed relative to fetal liver, a major site of expression and metformin uptake [3].

### Statistical Analyses

Metformin concentrations 24 h post-administration in maternal versus fetal artery were analyzed using ratio paired *t*-test. Transporter expression in cotyledon and caruncle tissue was normalized to expression in the fetal liver and analyzed with Wilcoxon signed rank test compared to a median of 1.0, representing fetal liver expression. Mean ± SE are shown. Significance was considered *P* < 0.05.

## Results

### Evaluation of Metformin in Maternal and Fetal Circulation

**Fig. 1:**
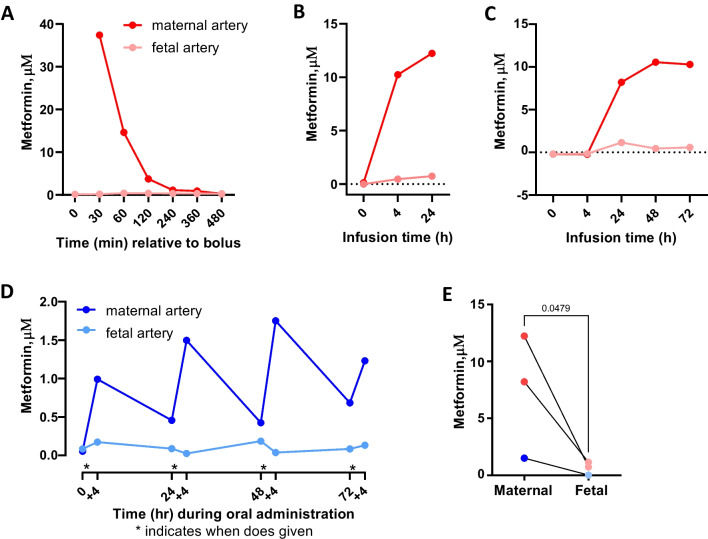
Metformin concentrations in maternal and fetal circulation. Maternal and fetal artery plasma concentrations of metformin were measured as indicated following different routes of maternal metformin administration (*n* = 1 each). A bolus (500 mg) of metformin was infused intravenously **A**. Metformin was infused continuously at 83 mg/h for 24 **B** or 72 h **C**. Metformin (2000 mg) was added to the drinking water of pregnant ewes once daily over 72 h **D**. Maternal compared to fetal artery plasma metformin concentrations (*n* = 3 animals with paired samples) measured at 24 h in panels **B** and **C** and 28 h in panel **D**. Ratio paired *t*-test and *p*-value shown **E**

A single 500 mg dose of metformin was administered to pregnant sheep. Although detectable in maternal plasma, metformin was undetectable in fetal plasma (Fig. 1A). Following continuous infusion, maternal metformin plasma concentrations reached ~ 12 μm within 24 h but were undetectable in fetal plasma (Fig. 1B). Continuous infusion was extended over 3 days. Metformin concentrations in maternal artery reached ~ 10 μM and remained constant over 3 days but were undetectable in fetal arterial plasma (Fig. 1C). Oral dosing increased maternal concentrations to 1-2 μM within 4 h after each dose, but fetal arterial concentrations were undetectable (Fig. 1D). Both intravenous and oral administration to pregnant ewe increased maternal arterial plasma concentrations of metformin but failed to produce detectable metformin in fetal blood (Fig. 1E).

### Metformin Transporter Expression in the Placenta

**Fig. 2:**
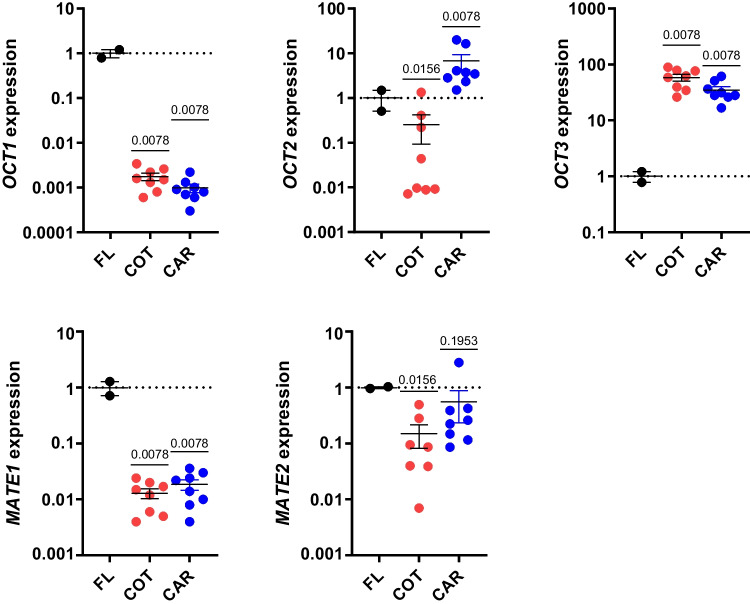
Metformin transporter expression in placental tissues. Gene expressions of *OCT1*, *OCT2*, *OCT3*, *MATE1*, and *MATE2* was measured in cotyledon (COT, *n* = 8) and caruncle (CAR, *n* = 8) tissue and compared to the fetal liver (FL, *n* = 2). COT and CAR expression was compared to FL expression with Wilcoxon signed rank test. Mean ± SE are shown. Significance was considered when *P* < 0.05

Expression of organic cation transporter (*OCT*)*1* in cotyledon and caruncle tissue was < 1% of the expression measured in fetal liver (Fig. 2). *OCT2* expression was 75% lower in cotyledon and sixfold higher in caruncle tissue compared to fetal liver. *OCT3* expression was ~ 50-fold higher in cotyledon and caruncle tissues compared to fetal liver. Multidrug and toxin extrusion proteins (*MATE*)*1* expression was lower in cotyledon and caruncle tissue compared to fetal liver. *MATE2* was lower in cotyledon and similar in caruncle tissue compared to fetal liver.

## Discussion

Our data show that metformin administered to pregnant ewe increases maternal concentrations, yet metformin is undetectable in the fetus even after 3–4 days of monitoring and using different administration routes. This is the first study showing that metformin does not cross ovine placenta and accumulate in the fetus. This contrasts with observations in humans and mice but is consistent with observations in chinchilla [4, 9]. Likewise, previous studies show that resveratrol crosses the placenta in humans and other species but does not cross ovine placenta [10]. The impermeability of the sheep placenta could reflect structural differences in the sheep compared to human placenta [11, 12] with increased interhaemal distance and more interdigitations between the maternal and fetal interface that prevent metformin transfer from mother to fetus.

In humans, *OCT1* expression and metformin uptake by the liver are required for its therapeutic effects on glucose homeostasis. Similarly, ovine and macaque fetal liver tissues and isolated hepatocytes have abundant *OCT1* expression [3]. The ovine fetal liver has metformin uptake following direct fetal metformin infusion that achieved human dose equivalence of ~ 40 μM [3]. We show that *OCT1* expression in placental cotyledon and caruncle tissue is < 1% of expression in fetal liver. *OCT2, OCT3, MATE1*, and *MATE2* are present in ovine placenta, though expression levels or localization of these transporters appear insufficient to mediate placental transport.

A major limitation of human and rodent studies is the inability to test and compartmentalize effects of metformin on the fetus independently of effects of maternal phenotype or placental influences. Since maternally administered metformin in sheep does not cross the placenta, yet human dose-equivalent levels of metformin can be reached using direct fetal infusion [3], pregnant sheep can be used as a model to “compartmentalize” and test direct effects of metformin on the fetus, apart from confounding maternal and placental influences. Conversely, pregnant sheep could be used to test the effects of metformin in only the maternal compartment.

## Data Availability

All data and materials used in this research are available upon request.
